# Fare well to Nova Scotia? Public health investments remain chronically underfunded

**DOI:** 10.17269/s41997-021-00478-8

**Published:** 2021-02-24

**Authors:** Hilary A. T. Caldwell, Sarah Scruton, Katherine Fierlbeck, Mohammad Hajizadeh, Shivani Dave, S. Meaghan Sim, Sara F. L. Kirk

**Affiliations:** 1 Healthy Populations Institute, Dalhousie University, 1318 Robie Street, Halifax, NS B3H 3E2 Canada; 2 Department of Political Science, Dalhousie University, 6299 South Street, Rm 301, PO Box 15000, Halifax, NS B3H 4R2 Canada; 3 MacEachen Institute of Public Policy and Governance, Dalhousie University, Macdonald Building, 2nd Floor, 6300 Coburg Road, PO Box 15000, Halifax, NS B3H 4R2 Canada; 4 School of Health Administration, Dalhousie University, Sir Charles Tupper Medical Building, 2nd Floor, 5850 College Street, PO Box 15000, Halifax, NS B3H 4R2 Canada; 5 Nova Scotia Health Authority Research, Innovation & Discovery, Centre for Clinical Research, Room 177, 5790 University Avenue, Halifax, NS B3H 1V7 Canada

**Keywords:** Public health, Health administration, Health system, Funding, Nova Scotia, Santé publique, administration sanitaire, système de santé, financement, Nouvelle-Écosse

## Abstract

Inspired by Fiset-Laniel et al.’s (2020) article entitled “Public health investments: neglect or wilful omission? Historical trends in Quebec and implications for Canada”, we assessed public health investments since the establishment of the Nova Scotia provincial health authority in 2015. We analyzed Nova Scotia Department of Health and Wellness budgets from 2015−2016 to 2019–2020 and observed that less than 1% of funding was budgeted for public health annually, an amount well below the recommendation that 5–6% of healthcare funding be spent on public health. Healthcare spending has increased annually since 2015–2016, but proportions of funding to different programs and services have remained static. Specifically, we did not observe a change in investment in public health over time, suggesting that while the government does not necessarily spend too much or too little on healthcare, it spends far too little on public health. This chronic under-funding is problematic given the high rates of non-communicable diseases in Nova Scotia and health inequities experienced within the population. The 2020 COVID-19 pandemic has highlighted the importance of public health work, and the need for a pandemic recovery plan that prioritizes investment in all areas of public health in Nova Scotia.

## Résumé

Inspirés par l’article de Fiset-Laniel et coll. (2020) intitulé « *Public health investments: neglect or wilful omission? Historical trends in Quebec and implications for Canada* », nous avons évalué les investissements en santé publique depuis la fondation de l’autorité sanitaire provinciale de la Nouvelle-Écosse en 2015. Nous avons analysé les budgets du ministère de la Santé et du Mieux-Être de la Nouvelle-Écosse de 2015−2016 à 2019−2020 et nous avons observé que moins de 1 % du financement était prévu pour la santé publique annuellement, un montant bien inférieur à la recommandation que 5−6 % du financement pour les soins de santé soit dépensé sur la santé publique. Les dépenses de santé ont augmenté annuellement depuis 2015−2016, mais les proportions du financement consacrés à différents programmes et services ont demeuré statiques. Spécifiquement, nous n’avons pas observé de changement dans l’investissement en santé publique au fil du temps, indiquant que tandis que le gouvernement ne dépense pas nécessairement trop ou trop peu sur les soins de santé, il dépense bien trop peu sur la santé publique. Ce sous-financement chronique est problématique étant donné les hauts taux de maladies non transmissibles en Nouvelle-Écosse et les inégalités en matière de santé qui existent au sein de la population. La pandémie de la COVID-19 de 2020 a souligné l’importance du travail lié à la santé publique, ainsi que la nécessité d’un plan de rétablissement suite à une pandémie qui priorise l’investissement dans tous les domaines de santé publique en Nouvelle-Écosse.

## Introduction

In “Public health investments: neglect or wilful omission? Historical trends in Quebec and implications for Canada”, Fiset-Laniel and colleagues describe the declining investment in public health in Quebec and highlight that adequate investment is needed in public health and social determinants of health to protect population health in that province (Fiset-Laniel et al. 2020). Inspired by Fiset-Laniel et al., we analyzed trends in public health investments in Nova Scotia (NS) from 2015 to 2020. Through this process, we suggest not only that the provision of public health in NS has suffered in the past 5 years, but also that the current COVID-19 crisis may undermine provincial public health capacity even further.

## Public health on life support

The Public Health Agency of Canada describes public health as a combination of programs, services and policies to keep people healthy and prevent injury, illness and premature death (Public Health Agency of Canada 2008). Research supports investment in public health interventions, such as health protection investments or legislative interventions, as they have a substantial return on investment and lead to future cost savings (Masters et al. 2017). Indeed, Guyon and Perreault (2016) suggest a 10% increase in public health spending will decrease mortality by 1.1–6.9%. Given the importance of public health efforts for disease prevention and addressing health inequities, and the documented return on investment, governments should be increasing, not decreasing, their investments in public health. Yet, Canadian public health investments have remained historically low at 5.4% of total healthcare expenditures, despite ongoing calls for increased federal and provincial expenditure (Canadian Institute for Health Information, 2019). Following the SARS outbreak in Canada in 2003–2004, provincial governments were urged to invest 5–6% of annual health budgets into public health (Canadian Institute for Health Information, 2019). Public health officials have been increasingly concerned with the marginalization of public healthcare and decreased funding for some years (Guyon et al. 2017); and, while COVID-19 has focused attention more sharply on public health per se (Goel 2020), it does not address the long-term structural issues associated with sustainable public health delivery.

**Fig. 1:**
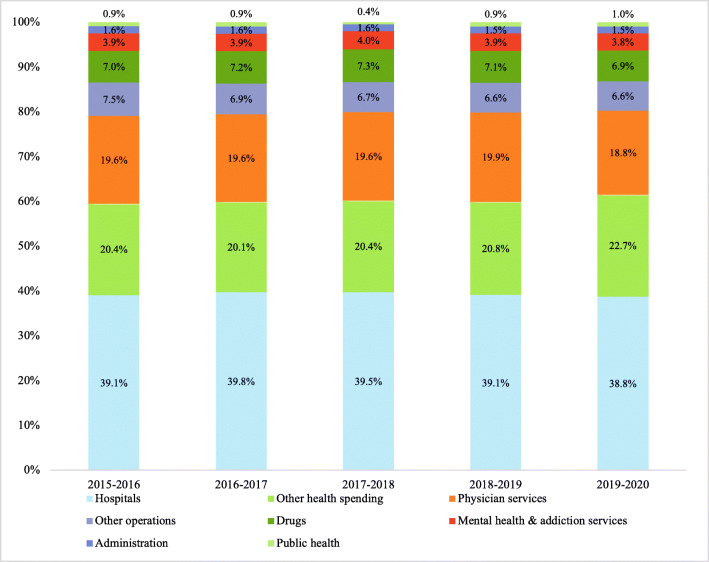
Provincial government health spending by category, 2015–2016 to 2019–2020. Data obtained from the Nova Scotia Department of Finance and Treasury Board

While British Columbia and Ontario have reached a minimum investment of 5–6%, NS has continued to under-invest in public health, and remains the province with the lowest investment in public health funding in Canada (Canadian Institute for Health Information 2019). As Fig. 1 shows, NS invests only 1% of its provincial health spending on public health. This is a policy decision that could have serious consequences for a province with a population frequently recognized as being among the least healthy in the country, coupled with observable health inequities within the population (Fierlbeck 2018; Hajizadeh et al. 2014).

## Trends in public health investments in NS

In 2019, 39% of NS provincial health spending funded hospitals, while 19% funded physicians and 1% funded public health (Nova Scotia Finance and Treasury Board 2020). Since 2000, hospital spending has declined and funding to other institutions increased, while public health spending remained static. This is especially problematic given the recommendations to increase public health spending following the SARS outbreak in 2003–2004 (Canadian Institute for Health Information 2019; Guyon et al. 2017). Provincial restructuring in 2015 to form the Department of Health and Wellness (DHW) and the two provincial health service and delivery entities (Nova Scotia Health [NSH] and the IWK Health Centre) was expected to fortify public health funding, but this has not materialized (Fierlbeck 2018). In NS, public health is delivered through NSH. To better understand historical and current public health spending, we reviewed and analyzed publicly available data from the Finance and Treasury Board - Province of Nova Scotia (Nova Scotia Finance and Treasury Board 2020). We determined that following the formation of NSH in 2015, public health spending has remained chronically low at less than 2% of the NSH budget, and the amount is still less than that in the year that NSH was formed (Nova Scotia Health Authority 2019). The high spending in hospital and physician services versus public health causes major gaps in the health system.

Trends in the Nova Scotia DHW budget from 2015–2016 to 2019–2020 are reported in Fig. 1 and Table 1. What this shows is that the government does not necessarily spend too much or too little on healthcare, but it spends far too little on public health. While the absolute value of the investment in health service and delivery has increased 12.1% from 2015–2016 to 2019–2020, there has been little change in the amount as a percentage of the Nova Scotia provincial budget. Public health has seen an annual increase of 3.3%, accumulating to an overall increase of 20% in absolute funding from 2015–2016 to 2019–2020 ($38,684,000 to $44,229,000; Table 1), but it has remained at less than 1% of the Department’s budget since 2015–2016 (Fig. 1). Overall, health spending rose by 2.1% annually from 2010 to 2018, but in 2019 it only increased by 1.5% (Canadian Institute for Health Information 2019). In 2006, the NS government was encouraged to more than double the 1.2% public health investment to 2.4% within a decade (Fierlbeck 2018). In addition, the less than 1% investment (Fig. 1) remains well below the suggested investment of 5% of healthcare spending per federal government recommendations (Moulton 2006). Despite the growing investments in the delivery of health services and supports in NS, public health has not benefited; the budget remains well below this recommendation (Canadian Institute for Health Information 2019; Hampton 2020). Astonishingly, the last time that the public health budget in NS met the recommended funding levels was in 1975 (Hampton 2020).

**Table 1:** Spending on health service programs in Nova Scotia, 2015–2016 to 2019–2020

Programs	Budget (in $1000) 2019–2020	% share from total health budget (2019–2020)	% change between 2015–2016 and 2019–2020	Average annual increase between 2015–2016 and 2019–2020	% change between 2018–2019 and 2019–2020
Administration	69,234	1.49%	+ 4.14%	+ 0.69%	+ 2.56%
Other operations	304,573	6.57%	− 2.18%	− 0.36%	+ 5.19%
Physician services	870,839	18.77%	+ 7.63%	+ 1.27%	+ 0.14%
Drugs	318,812	6.87%	+ 10.22%	+ 1.70%	+ 2.44%
Hospitals	1,799,733	38.80%	+ 11.22%	+ 1.87%	+ 5.29%
Public health	44,229	0.95%	+ 19.98%	+ 3.33%	+ 8.53%
Mental health and addiction services	176,955	3.81%	+ 9.85%	+ 1.64%	+ 4.08%
Other health spending	1,054,151	22.73%	+ 24.70%	+ 4.12%	+ 15.94%
Total budget – NS Health and Wellness	4,638,526	100.00%	+ 12.10%	+ 2.02%	+ 6.22%
Total investments in Nova Scotia	10,101,784		+ 13.38%	+ 2.23%	+ 4.21%

Data obtained from the Nova Scotia Department of Finance and Treasury Board

When the public health system is faced with a public health emergency like the COVID-19 pandemic, the capacity deficits result in diminished services as resources are re-allocated to the pandemic response. This leads to a decline in other essential public health services, such as school vaccination clinics (Canadian Institute for Health Information 2019). Although funding for hospitals, pharmaceuticals and physicians are essential expenditures, the lack of financial resources allocated to public health weakens preventive care despite evidence that it is vital to preserving and protecting population health (Guyon and Perreault 2016). Reports have highlighted the importance of public health to tackle COVID-19 and that perhaps this can be an opportunity for further public health reform to improve population health outcomes (Fierlbeck 2018). In fact, increased financial resources, as one of the main structural elements, have been shown to improve performance of public health systems (Guyon and Perreault 2016).

What legacy will COVID-19 have on public health once the pandemic recedes? Will it lead to a more robust health system? In 2002, NS was the only province to have a Department of Health Promotion and Protection, separate from the Department of Health. In 2003, the SARS crisis exposed structural deficiencies in Ontario’s health system. To avoid similar deficiencies in NS, the DHW was established in 2012 to replace the two previous health-focused departments. Meanwhile, in response to the H1N1 pandemic in 2009, NS negotiated “a good neighbour protocol” between healthcare unions to allow staff to move between various organizations to fill shortages associated with public health emergencies (Fierlbeck 2018). This protocol was used in May 2020 to redeploy NSH staff to areas in which staff were most needed, including long-term care facilities (North American Observatory on Health Systems and Policies 2020). These two pandemic responses have forever changed public health in NS as it became more administratively connected with healthcare. COVID-19 has the potential to elicit further change and a pandemic recovery plan needs to prioritize public health in order to address the widespread prevalence of non-communicable diseases and health inequities. A recent rapid review suggests that community-based public health measures in pandemics, including H1N1 and COVID-19, can have negative effects on children and youth, including decreased vaccination rates as a result of school closures and decreased physical activity participation as a result of stay-at-home recommendations (Ontario Agency for Health Protection and Promotion (Public Health Ontario) 2020). It is understood that these measures are necessary to control the spread of infectious diseases and there is no doubt that public health is essential to the pandemic response. But future investment in public health, beyond the immediacy of communicable disease mitigation, is needed post-pandemic to ensure Nova Scotians have access to essential public health services that promote health and address inequities. As it stands, the current investment in public health is too low to meet this goal.

## Conclusion

Our findings build support for a pan-Canadian policy response to address the longstanding challenges of a chronically underfunded public health system. Based on provincial healthcare budgets and current spending, NS is continually in last place for public health spending among Canadian provinces. The province should be commended on its response to the COVID-19 pandemic, but this should not be used to justify continued inadequate investment in public health. The province’s response has been possible at the expense of regular public health services and supports that will need to be prioritized as we recover from the pandemic so as to address the burden of non-communicable diseases. The COVID-19 pandemic has highlighted our province’s public health heroes, and increased and sustained investment in public health in Nova Scotia, in its broadest sense, should be part of COVID-19’s legacy.

## Data Availability

This analysis used publicly available data from the Nova Scotia Treasury Board.
